# The Effect of Ondansetron in Prevention of Postoperative Shivering after General Anesthesia in Gynecological Surgery

**Published:** 2012-05-30

**Authors:** M Entezari Asl, Kh Isazadehfar, G Akhavanakbari, M Khoshbaten

**Affiliations:** 1 Department of Anesthesiology, Ardabil University of Medical Sciences, Ardebil, Iran; 2 Department of Community and Health Medicine, Ardabil University of Medical Sciences, Ardebil, Iran; 3 Department of Gasteroenterology, Tabriz University of Medical Sciences, Tabriz, Iran

**Keywords:** General anesthesia, Postoperative shivering, Meperidine, Ondansetron

Dear Editor,

Shivering happens frequently in 5 to 60% of surgical operations.[1] Though it occurs as a result of temperature reduction, it may occur in patients with a post operative normal body temperature.[2] Shivering may be dangerous due to increasing effect of medicines, extension of recovery period, increased oxygen consumption or hemostatic dysfunction especially in patients with a low cardiac reserve.[3]

Several medications have been suggested for prevention and treatment. According to regulator effect of intra-hypothalamic serotonin (5-ydroxytryptamine), its agonists (ondansetron) are reported to have the same effect.[4] Opposed to meperidine, ondansetron may reduce post-operative nausea and vomiting (PONV).[5]

A medication to reduce both PONV and shivering will be very valuable.[6] This study evaluated preventive effect of ondansetron and meperidine on post operative shivering compared to a control group.

We did this study in Alavi University Hospital of Ardabil (IRAN) in 2008. The protocol was approved by ethical committee of this university. All patients were in ASA physiologic class I or II. Patients were randomly selected to receive either 4 mg ondansetron, 0.4 mg/kg meperidine or 2 ml normal saline (the placebo) 2 minutes before inducting anesthesia.

We induced anesthesia by 1µg/kg fentanyl, 5 mg/kg thiopental and 1 mg/kg succinylcholine continued by 0.8-1% isoflurane along with the inspiration mixed of 50% oxygen and 50% N2O after intubation. Room temperature was set at 20-22°C.

We measured core body and skin temperature through tympanic membrane and forehead accordingly. Patients were observed for shivering (chills for at least 15 seconds), nausea or vomiting during recovery by an anesthesiologist blinded to the medication. Patients received appropriate care by meperidine for shivering and metoclopramide for nausea or vomiting when necessary.

Data were statistically analyzed using SPSS software (version 15, Chicago, IL, USA). All data were expressed as the mean±SD. A repeated-measure ANOVA was used for comparing core body temperature, skin temperature, systolic and diastolic blood pressure and heart rate in several stages (basic, post induction of anesthesia, end of surgery and in recovery room) in each group and Chi-Square test wase used for comparing the prevalence of shivering, nausea and vomiting in each group. A p-value less than 0.05 was considered significant.

There was no significant difference in age (p=0.49), operation duration (p=0.34), core body temperature (p=0.76) and skin temperature (p=0.79) between the groups. Core body temperature decline in control group was more in patients receiving ondansetron or meperidine (p=0.001), but no significant difference was observed between ondansetron and meperidine groups (p=0.09). There was no difference among three groups in the skin temperature decrease (p=0.27).

Systolic and diastolic blood pressure was decreased in all of patients during the surgery and back to basic value in recovery. Heart rate increased after induction of anesthesia and decreased slightly during the operation and recovery. No significant difference [Systolic blood pressure (p=0.66), diastolic blood pressure (p=0.42) and heart rate (p=0.81)] was noticed between three groups (p>0.05).

Shivering occurred significantly lower in patients receiving ondansetron or meperidine compared whit control group (p value=0.003). Results are shown in Table 1.

**Table 1:** Results of post-operative observation in three groups.

****	**Control group (%)**	**Ondansetron (%)**	**Meperidine (%)**	***P value***
Shivering	15 (50.0)	4 (13.3)	6 (20.0)	0.003
Nausea	3 (10.0)	3 (10.0)	3 (10.0)	1.00
Vomiting	2 (6.7)	0	1 (3.3)	0.30

The current study reports valuable preventive effects on shivering for ondansetron and meperidine compared to controls compatible with some previous reports. Kelsaka reported a reduction in occurrence of shivering after a spinal anesthesia from 36% (in controls) to 8% by either ondansetron or meperidine.[4] Piper reported failure of 12.5 mg dolansetron to decrease this rate which may be due to its inadequate dosage.[1] While Powel reported shivering to occur in 57% of patients receiving saline compared to a rate of 33% followed by 4 mg ondansetron (15%) followed by 8 mg ondansetron.[7] They highlighted the effect of ondansetron to interfere thermoregulation by a central mechanism.

Although meperidine has the minority effect on cardiovascular system with the dosage used for treating shivering (0.3-0.4 mg/kg), it may extensively depress respiration especially if used during the surgery.[8] Results of this study demonstrated the safety of using ondansetron as well as its efficacy.

In conclusion using ondansetron instead of meperidine is suggested because of its ability to reduce shivering from 50 to 13.3% in addition to lower side effects especially in patients with homodynamic instability.
